# Hepatic MiR-291b-3p Mediated Glucose Metabolism by Directly Targeting p65 to Upregulate PTEN Expression

**DOI:** 10.1038/srep39899

**Published:** 2017-01-05

**Authors:** Jun Guo, Lin Dou, Xiangyu Meng, Zhenzhen Chen, Weili Yang, Weiwei Fang, Chunxiao Yang, Xiuqing Huang, Weiqing Tang, Jichun Yang, Jian Li

**Affiliations:** 1 The MOH Key Laboratory of Geriatrics, Beijing Hospital, National Center of Gerontology, Beijing, 100730, P. R. China; 2 Department of physiology and pathophysiology, key laboratory of molecular cardiovascular science of the ministry of education, Peking University Health Science Center, Beijing 100191, China

## Abstract

Several studies have suggested an important role of miR-291b-3p in the development of embryonic stem cells. In previous study, we found that the expression of miR-291b-3p was significantly upregulated in the liver of db/db mice. However, the role of miR-291b-3p in glucose metabolism and its underlying mechanisms remain unknown. In the present study, we demonstrated that miR-291b-3p was abundantly expressed in the liver. Of note, hepatic miR-291b-3p expression was upregulated in HFD-fed mice and induced by fasting in C57BL/6 J normal mice. Importantly, hepatic inhibition miR-291b-3p expression ameliorated hyperglycemia and insulin resistance in HFD-fed mice, whereas hepatic overexpression of miR-291b-3p led to hyperglycemia and insulin resistance in C57BL/6 J normal mice. Further study revealed that miR-291b-3p suppressed insulin-stimulated AKT/GSK signaling and increased the expression of gluconeogenic genes in hepatocytes. Moreover, we identified that p65, a subunit of nuclear factor-κB (NF-κB), is a target of miR-291b-3p by bioinformatics analysis and luciferase reporter assay. Silencing of p65 significantly augmented the expression of PTEN and impaired AKT activation. In conclusion, we found novel evidence suggesting that hepatic miR-291b-3p mediated glycogen synthesis and gluconeogenesis through targeting p65 to regulate PTEN expression. Our findings indicate the therapeutic potential of miR-291b-3p inhibitor in hyperglycemia and insulin resistance.

Throughout the world, type-2 diabetes (T2D) is a major health concern that is affecting not only in adults but also in children and this disease is increasing at an alarming rate^1^,^2^. Genetic factors, obesity, and lifestyle factors are widely accepted as the major contributors to this disease^3^,^4^. Moreover, it is well established that T2D is the principal consequence of insulin resistance^5^. Insulin resistance is defined as a diminished capacity of targeted cells, such as adipocytes, skeletal muscle cells, and hepatocytes, to respond to insulin^6^. Insulin resistance in the liver leads to impaired glycogen synthesis and failure to suppress glucose production. However, we still lack knowledge about the underlying molecular mechanism for hepatic insulin resistance.

The phosphatidylinositol 3 kinase (PI3K)/serine/threonine kinase (AKT) signaling pathway is critical for the body to maintain numerous crucial cellular functions, including glucose metabolism, cell proliferation and apoptosis^7^,^8^. Inactivation of the PI3K/AKT signaling has been considered as a hallmark for metabolic diseases^9^. For the liver, aberrant PI3K/AKT signaling has increasingly emerged as one of the major reasons for liver dysfunction and the metabolic syndrome^10^. A number of external stimuli and substantial regulators were indicated to regulate the activity of PI3K/ AKT signaling^11^. Among them, phosphatase and tensin homolog deleted on chromosome 10 (PTEN) antagonizes PI3K actions to negatively regulate the phosphorylation of AKT. Numerous studies revealed that overexpression of PTEN could suppress insulin-induced PtdIns (3,4) P2/PtdIns (3,4,5) P3 PIP3 production, thereby inhibiting AKT activation, GLUT4 translocation and glucose uptake^12^,^13^,^14^.

PTEN expression levels are under strict monitoring at the transcriptional level and the post-translational level. At present, several transcription factors and pathways were identified to regulate the expression of PTEN. For example, p38 mitogen-activated protein kinases (MAPK) was found to increase the protein expression of PTEN in human aortic vascular endothelial cells and transforming growth factor (TGF)-β was demonstrated to suppress PTEN transcription in pancreatic cancer cells^15^,^16^. Furthermore, the transcription of PTEN was also found to be inhibited by JUN, a proto-oncogenic transcription factor, in multiple human tumor cell lines^17^. Notably, in a panel of cancer cells, p65, a subunit of nuclear factor-κB (NF-κB), could repress the expression of PTEN, thereby prompting tumor growth through the PI3K/AKT pathway^18^,^19^. Thus, dysregulation of PTEN expression represents a potential therapeutic for many diseases, including cancer and metabolic syndrome.

MicroRNAs (miRs) are small, non-coding RNAs that exert biological effects by translational repression or degradation of target messenger RNAs (mRNAs)^20^. It was estimated that the expression of ~60% of genes is modulated by mature miRNAs^21^. Recently, it is implicated that aberrant expression of miRs is associated with insulin resistance. In previous study, we found several miRs to be involved in insulin resistance. For example, miR-200s contributed to IL-6-induced insulin resistance in hepatocytes^22^. MiR-19a regulated PTEN expression to mediate glycogen synthesis in hepatocytes^23^. In addition, we demonstrated that the expression of miR-291b-3p was significantly upregulated in db/db and HFD-fed mice. However, the functional role of miR-291b-3p in hepatic insulin resistance has not been elucidated. Here, we explored the role of miR-291b-3p in regulating insulin signaling and glucose metabolism. We found novel evidence suggesting that hepatic miR-291b-3p mediated glycogen synthesis and gluconeogenesis through targeting p65 to regulate PTEN expression.

## Results

### Hepatic miR-291b-3p expression is upregulated in HFD-fed mice and induced by fasting in C57BL/6 J normal mice

**Figure 1:**
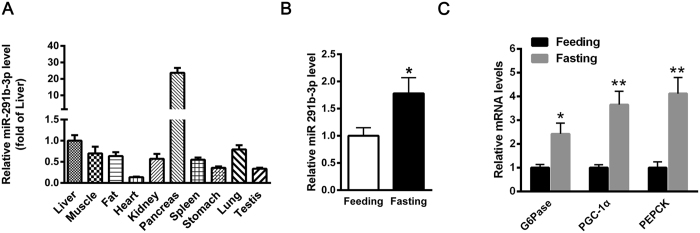
Hepatic miR-291b-3p expression is upregulated in HFD-fed mice and induced by fasting in C57BL/6 J normal mice. (**A**) The expression of miR-291b-3p in different organs of C57BL/6 J normal mice, as analyzed by real-time PCR. (**B**) The expression of miR-291b-3p in the liver of C57BL/6 J mice fasted for 16 h. (**C**) The mRNA levels of G6Pase, PGC1-α and PEPCK were determined in the liver of fasted mice. Data are expressed as mean ± SEM (n = 5). *P < 0.05; **P < 0.01, ***P < 0.001 vs. other group.

In previous study, we demonstrated that the expression level of miR-291b-3p was increased in the liver of db/db mice and HFD-fed mice. In this study, we further profiled the expression of miR-291b-3p in different organs. As analyzed by real-time PCR, miR-291b-3p is widely expressed in main organs of C57BL/6 J mice including liver, muscle, fat, heart, kidney, pancreas, spleen, stomach, lung and testis. Of note, miR-291b-3p is abundantly expressed in the liver and the pancreas (Fig. 1A). Moreover, to explore whether the nutritional status could affect the expression pattern of hepatic miR-291b-3p, the level of miR-291b-3p was determined in the liver of C57BL/6 J mice fasted for 16 h. As shown in Fig. 1B, hepatic miR-291b-3p expression level was also increased in the fasted states. Meanwhile, fasting resulted an increase in mRNA levels of gluconeogenic genes including glucose-6-phosphatase (G6Pase), peroxisome proliferator-activated receptor gamma coactivator-1 alpha (PGC-1α), and phosphoenolpyruvate carboxykinase (PEPCK) (Fig. 1C) in the livers. These results imply that miR-291b-3p may be involved in hepatic insulin resistance.

### Hepatic inhibition miR-291b-3p expression ameliorates hyperglycemia and insulin resistance in HFD-fed mice

**Figure 2:**
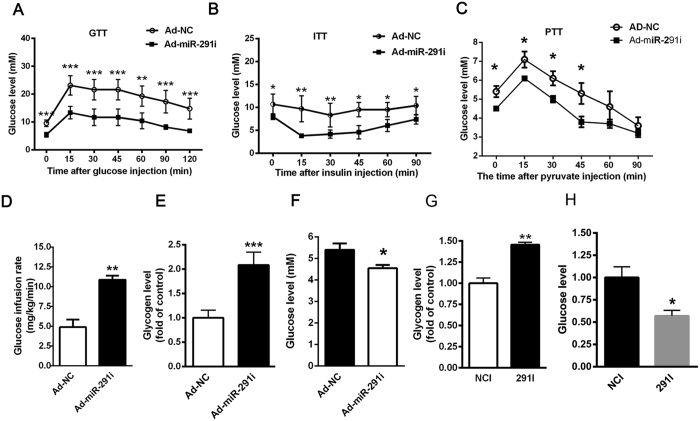
Hepatic inhibition miR-291b-3p expression ameliorates hyperglycemia and insulin resistance in HFD-fed mice. (**A**) Glucose tolerance test (GTT) in HFD-fed mice on 7 days after injection of adenovirus-miR-291b-3p-inbibitor (Ad-miR-291i) or adenovirus-GFP (Ad-NC). (**B**) Insulin tolerance test (ITT) in HFD-fed mice on 7 days after injection of Ad-miR-291i or Ad-NC. (**C**) Pyruvate tolerance test (PTT) in HFD-fed mice on 7 days after injection of Ad-miR-291i or Ad-NC. (**D**) Euglycemic-hyperinsulinemic clamp assay in HFD-fed mice on 7 days after injection of Ad-miR-291i or Ad-NC. (**E**) The glycogen content in the liver of HFD-fed mice on 7 days after injection of Ad-miR-291i or Ad-NC. (**F**) The glucose production in the liver of HFD-fed mice on 7 days after injection of Ad-miR-291i or Ad-NC. (**G**) The glycogen content in the NCTC1469 cells transfected with miR-291b-3p inhibitor (291i). (**H**) The glucose level in the medium of NCTC1469 cells transfected with 291i. Data are expressed as mean ± SEM (n = 5). *P < 0.05; **P < 0.01 vs. control group.

To directly assess the pathological role of miR-291b-3p in glucose metabolism and insulin resistance, recombinant adenovirus expressing miR-291b-3p inhibitor (ad-miR-291i) was injected into HFD-fed mice through tail vein. We previously identified that the hepatic levels of miR-291b-3p were decreased by ~40%, accompanied by a significant reduction in the liver weight and the liver weight-to-body weight ratio, and a significant decrease in hepatic lipid deposition^24^. In the present study, compared with ad-GFP-treated mice (ad-NC), the HFD-fed mice injected with ad-miR-291i exhibited a significant reduction in fasting blood glucose levels. Glucose tolerance was also significantly improved by hepatic inhibition miR-291b-3p expression, as measured by oral glucose tolerance test (Fig. 2A). In addition, adenovirus-mediated knockdown of miR-291b-3p improved insulin-sensitive performance, as identified by steeper rate of reduced blood glucose levels in response to insulin (Fig. 2B). A pyruvate tolerance test (PTT) clearly indicated that inhibition of miR-291b-3p repressed hepatic glucose production (Fig. 2C). Hyperinsulinemic euglycemic clamp assay demonstrated that inhibition of miR-291b-3p significantly improved the global insulin sensitivity of HFD-fed mice, as assessed by increased glucose infusion rates (Fig. 2D). Furthermore, silencing of miR-291b-3p in the liver of HFD-fed mice elevated glycogen level and reduced glucose production (Fig. 2E and F).

Next, we determined whether the glucose-lowering effects of miR-291b-3p inhibition is cell-autonomous, murine liver cell lines NCTC1469 was transfected with miR-291b-3p inhibitor. Consistent with the results *in vivo*, silencing of miR-291b-3p in NCTC1469 cells enhanced glycogen synthesis and suppressed glucose production (Fig. 2G and H). Taken together, our data suggest that hepatic inhibition miR-291b-3p expression ameliorated hyperglycemia and insulin resistance *in vivo* and *in vitro*.

### Hepatic overexpression of miR-291b-3p results in hyperglycemia and insulin resistance in C57BL/6 J mice

**Figure 3:**
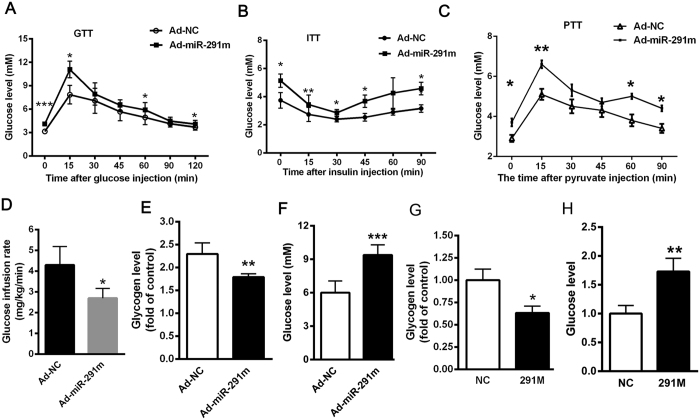
Hepatic overexpression of miR-291b-3p results in hyperglycemia and insulin resistance in C57BL/6 J mice. (**A**) GTT in C57BL/6 J mice on 7 days after injection of adenovirus- miR-291b-3p-mimic (Ad-miR-291m) or adenovirus-GFP (Ad-NC). (**B**) ITT in C57BL/6 J mice on 7 days after injection of Ad-miR-291m or Ad-NC. (**C**) PTT in C57BL/6 J mice on 7 days after injection of Ad-miR-291m or Ad-NC. (**D**) Euglycemic-hyperinsulinemic clamp assay in C57 mice on 7 days after injection of Ad-miR-291m or Ad-NC. (**E**) The glycogen content in the liver of C57BL/6 J mice on 7 days after injection of Ad-miR-291m or Ad-NC. (**F**) The glucose production in the liver of C57BL/6 J mice on 7 days after injection of Ad-miR-291m or Ad-NC. (**G**) The glycogen content in the NCTC1469 cells transfected with miR-291b-3p mimic (miR-291m). (**H**) The glucose level in the medium of NCTC1469 cells transfected with miR-291m. Data are expressed as mean ± SEM (n = 5). *P < 0.05; **P < 0.01; ***P < 0.001 vs. control group.

To gain further insights into the significance of miR-291b-3p in manipulating glucose metabolism and insulin resistance, recombinant adenovirus expressing miR-291b-3p mimic was injected into C57BL/6 J normal mice through tail vein. Adenovirus-mediated overexpression of miR-291b-3p in C57BL/6 J mice led to impaired glucose and insulin tolerance, as indicated by GTT and ITT experiments (Fig. 3A and B). Similarly, overexpression of miR-291b-3p in the liver of C57BL/6 J mice significantly increased de novo hepatic glucose production (Fig. 3C). As expected, ad-miR-291m-treated mice exhibited a decrease in hepatic glucose infusion rate, glycogen content and an increase in glucose level (Fig. 3D,E and F). Moreover, when we examined glycogen synthesis and glucose production in NCTC1469 cells, we found a significant reduction of glycogen content and a striking increase in glucose production by miR-291b-3p overexpression (Fig. 3G and H). Based on these observations, we proposed that miR-291b-3p, as a liver-enriched miRNA, could prompt hepatic insulin resistance in mice.

### MiR-291b-3p suppresses insulin-stimulated AKT/GSK/FoxO1 signaling and increases the expression of gluconeogenic genes in hepatocytes

**Figure 4:**
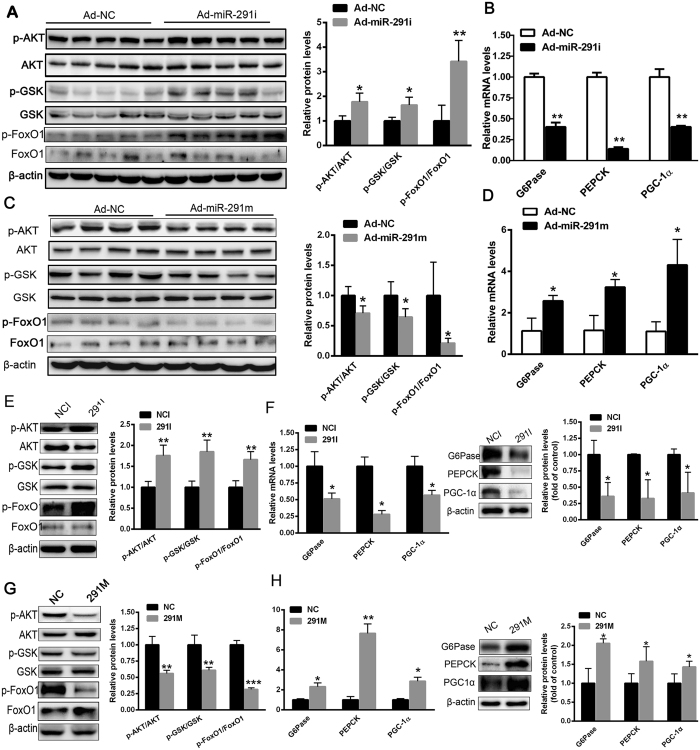
MiR-291b-3p suppresses insulin-stimulated AKT/GSK/FoxO1 signaling and increases expression of gluconeogenic genes in hepatocytes. (**A**) The phosphorylation of AKT/GSK/FoxO1 in the liver of HFD-fed mice on 7 days after injection of Ad-miR-291i or Ad-NC. Full-length blots/gels are presented in Supplementary Figure 1. (**B**) The mRNA levels of gluconeogenesis genes including G6Pase, PGC-1α and PEPCK in the liver of HFD-fed mice on 7 days after injection of Ad-miR-291i or Ad-NC. (**C**) The phosphorylation of AKT/GSK/FoxO1 in the liver of C57BL/6 J mice on 7 days after injection of Ad-miR-291m or Ad-NC. Full-length blots/gels are presented in Supplementary Figure 2 and cropping lines are indicated in red color. (**D**) The mRNA levels of G6Pase, PGC-1α and PEPCK in the liver of C57BL/6 J mice on 7 days after injection of Ad-miR-291m or Ad-NC. (**E**) The phosphorylation of AKT/GSK/FoxO1 in the NCTC1469 cells transfected with miR-291b-3p inhibitor (291i). Full-length blots/gels are presented in Supplementary Figure 3 and cropping lines are indicated in red color. (**F**) The mRNA and protein levels of gluconeogenesis genes in the NCTC1469 cells transfected with 291i. (**G**) The phosphorylation of AKT/GSK/FoxO1 in the NCTC1469 cells transfected with miR-291b-3p mimic (291 m). Full-length blots/gels are presented in Supplementary Figure 4 and cropping lines are indicated in red color. (**H**) The mRNA and protein levels of G6Pase, PGC-1α and PEPCK in the NCTC1469 cells transfected with 291 m. Data are expressed as mean ± SEM (n = 5). *P < 0.05; **P < 0.01 vs. control group.

We next explored the underlying mechanism for the ameliorated hyperglycemia and hepatic insulin resistance in HFD-fed mice injected with ad-miR-291i. As shown in Fig. 4A, hepatic inhibition miR-291b-3p expression in HFD-fed mice elevated the phosphorylation levels of AKT/glycogen synthase kinase (GSK)/Forkhead box protein O1(FoxO1), whereas reduced the mRNA levels of gluconeogenic genes including G6Pase, PGC-1α, and PEPCK (Fig. 4B). In contrast, overexpression of miR-291b-3p in the liver of C57BL/6 J mice impaired the activation of AKT/GSK/FoxO1 signaling (Fig. 4C) and increased the transcript levels of G6Pase, PGC-1α and PEPCK (Fig. 4D). We further assessed the effect of miR-291b-3p on insulin-stimulated AKT/GSK/FoxO1 activation in cultured NCTC1469 hepatocytes. The NCTC1469 cells transfected with miR-291b-3p inhibitor exhibited increased AKT and GSK activity after insulin stimulation (Fig. 4E). Real-time PCR and Western blot analysis showed that silencing of miR-291b-3p suppressed the mRNA and protein levels of gluconeogenic genes (Fig. 4F). In contrast, following transfection with miR-291b-3p mimic in NCTC1469 cells, the activation of AKT/GSK/FoxO1 signaling was significantly lowered (Fig. 4G). Moreover, overexpression of miR-291b-3p caused an increase in mRNA and protein levels of G6Pase, PGC-1α and PEPCK (Fig. 4H). Taken together, these results demonstrate that miR-291b-3p contributed to hyperglycemia and hepatic insulin resistance through suppressing insulin-stimulated AKT/GSK signaling activation and increasing expression of gluconeogenic genes in hepatocytes.

### MiR-291b-3p elevates the expression of PTEN

**Figure 5:**
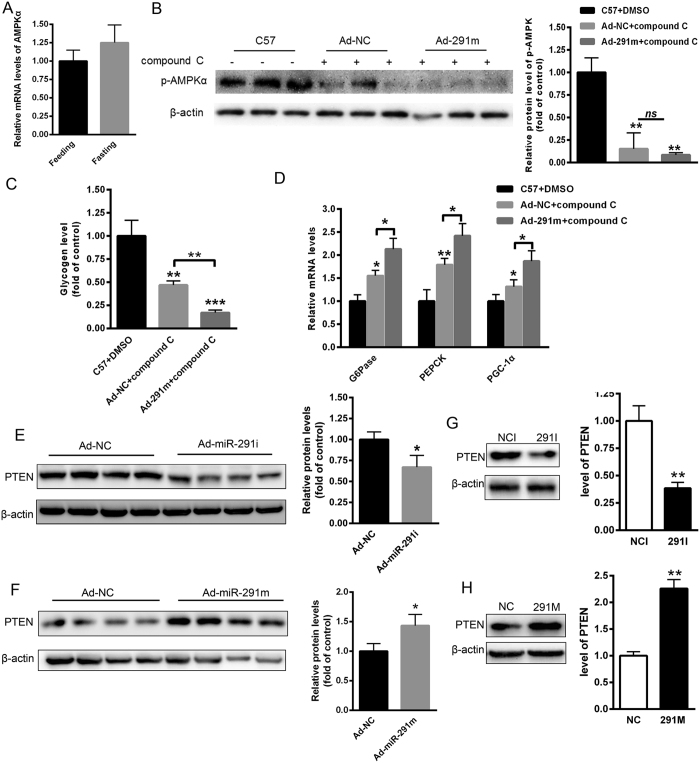
MiR-291b-3p elevates the expression of PTEN. (**A**) The mRNA level of AMPK in the liver of fasted mice. (**B**) The hepatic phosphorylation level of AMPK in the C57 mice injected with compound c, an AMPK inhibitor, at a dose of 2 mg/kg for 3 days. (**C**) The glycogen level in the liver of C57 mice transfected with Ad-NC and Ad-291m, and injected with compound C. (**D**) The mRNA levels of G6Pase, PGC1-α and PEPCK in the liver of C57 mice transfected with Ad-NC and Ad-291m, and injected with compound C. (**E**) The hepatic level of PTEN protein in the liver of HFD-fed mice on 7 days after injection of Ad-miR-291i or Ad-NC. (**F**) The hepatic expression of PTEN protein in the liver of C57BL/6 J mice on 7 days after injection of Ad-miR-291i or Ad-NC. (**G**) The protein level of PTEN in the NCTC1469 cells transfected with 291i or NC. (**H**) The expression of PTEN protein in the NCTC1469 cells transfected with 291 m or NC. Full-length blots/gels are presented in Supplementary Figure 5 and cropping lines are indicated in red color. Data are expressed as mean ± SEM (n = 5). *P < 0.05; **P < 0.01 vs. control group.

Next, we sought to determine how miR-291b-3p regulated AKT/GSK signaling activity. In our previously study, we identified AMPK as a primary target of miR-291, and revealed that miR-291 inhibitors ameliorated hepatic TG accumulation, reduced FAS and SREBP-1 activity and this was attributed to the targeting of miR-291b-3p of AMPK^24^. In the present study, we found that short-term fasting led to increased level of miR-291b-3p (Fig. 1B) without reduced expression of AMPK (Fig. 5A), indicating a different mechanism for glucogenesis under fasting. Moreover, to assess whether the effect of miR-291 on insulin resistance is dependent on AMPK, compound c (Sigma), an AMPK inhibitor, was intravenously injected at a dose of 2 mg/kg for 3 days before sacrifice. As shown in Fig. 5B, the phosphorylation level of AMPK was significantly suppressed by compound c, and no significant difference was found between ad-NC group and ad-miR-291m group after injection of compound c. Importantly, after inhibition of AMPK, overexpression of miR-291b-3p still significantly reduced hepatic glycogen level compared that of Ad-NC mice (Fig. 5C). Meanwhile, the levels of G6Pase, PGC-1α and PEPCK were significantly increased in the liver of Ad-291m mice even after suppression of AMPK activity (Fig. 5D). Therefore, we searched for another target of miR-291b-3p. PTEN has been demonstrated to negatively regulate the phosphorylation of AKT^25^. Given that PTEN is the major regulator controlling insulin resistance, we firstly analyzed the PTEN protein level in the liver of HFD-fed and chow-diet C57BL/6 J mice injected with ad-miR-291i or ad-miR-291m, respectively. Notably, silencing of miR-291b-3p in the liver of HFD-fed mice reduced hepatic expression of PTEN protein (Fig. 5E), while hepatic overexpression of miR-291b-3p in C57BL/6 J mice up-regulated PTEN expression (Fig. 5F). Accordingly, inhibition of miR-291b-3p resulted in a significant reduction of PTEN expression in NCTC1469 cells (Fig. 5G). However, overexpression of miR-291b-3p led to an increase in PTEN expression in NCTC1469 cells (Fig. 5H). These data indicate that hepatic increased PTEN expression might be involved in miR-291b-3p-reduced AKT/GSK signaling activity.

### MiR-291b-3p directly targets p65 to upregulate PTEN expression

**Figure 6:**
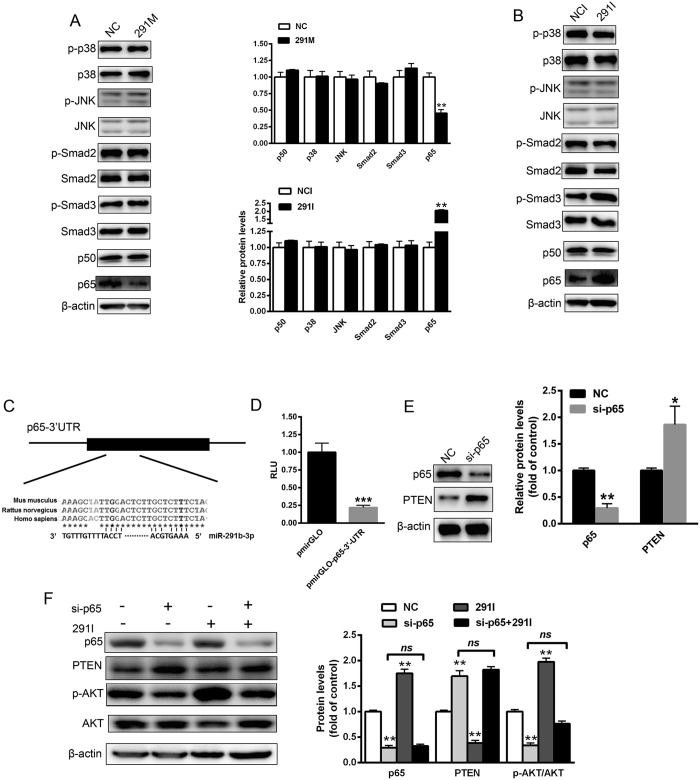
MiR-291b-3p directly targets p65 to upregulate PTEN expression. (**A**) The expression or activation of transcription factors and pathways regulating PTEN expression, including p38MAPK, JNK, Smad2/3 and NF-κB (p50 and p65) in the NCTC1469 cells transfected with 291 m or NC. Full-length blots/gels are presented in Supplementary Figure 6 and cropping lines are indicated in red color (left pannel). (**B**) The expression or activation of p38MAPK, JNK, Smad2/3 and NF-κB (p50 and p65) in the NCTC1469 cells transfected with 291i or NC. Full-length blots/gels are presented in Supplementary Figure 6 and cropping lines are indicated in red color (right pannel). (**C**) A conserved binding site of the 3′UTR of p65 with miR-291b-3p. (**D**) Relative luciferase activity in the NCTC1469 cells transfected with reporter constructs containing the 3′UTR of mouse p65 gene. (**E**) The levels of p65 and PTEN protein in the NCTC1469 cells transfected with a specific siRNA targeting p65. Full-length blots/gels are presented in Supplementary Figure 7A. (**F**) The levels of p65, PTEN, AKT and AKT phosphorylation in the NCTC1469 cells transfected with p65, miR-291b-3p inhibitor alone or both. Full-length blots/gels are presented in Supplementary Figure 7B and cropping lines are indicated in red color. Data are expressed as mean ± SEM (n = 3 independent experiments). *P < 0.05; **P < 0.01 vs. control group.

We further identified the direct effector of miR-291b-3p-induced increased PTEN expression. Several transcription factors and pathways including p38MAPK, JNK, Smad2/3 and NF-κB have been found to be involved in the regulation of PTEN transcription^18^. Firstly, we analyzed the effect of miR-291b-3p on these transcription factors and pathways. Strikingly, concomitant with increased PTEN expression, a converse decrease in p65 expression was observed in the NCTC1469 cells overexpressing miR-291b-3p (Fig. 6A). In contrast, inhibition of miR-291b-3p resulted in enhanced protein level of p65, accompanied by decreased PTEN expression (Fig. 6B). However, overexpression or inhibition of miR-291b-3p failed to affect the expression and activation of p38 MAPK, JNK and Smad2/3. Then, we aligned the 3′UTR of p65 with miR-291b-3p and identified a conserved binding site (Fig. 6C). To verify that p65 is a true target of miR-291b-3p, we generated dual luciferase reporter plasmids containing miR-291b-3p binding sites in the 3′UTRs of mouse p65. Luciferase reporter assay demonstrated that miR-291b-3p significantly decreased the luciferase reporter activity of pmirGLO-p65-3′UTR (Fig. 6D). To further verify that p65 negatively regulated the expression of PTEN, a specific siRNA targeting p65 was selected. Our data showed that silencing of p65 in NCTC1469 cells significantly increased the protein level of PTEN (Fig. 6E). More importantly, we found that inhibition of miR-291b-3p could not abolish p65 silencing-induced PTEN upregulation as well as impaired AKT activation (Fig. 6F). Taken together, these data suggest that miR-291b-3p directly targets p65 to upregulate PTEN expression, thereby antagonizing AKT/GSK activation.

## Discussion

Previously, we have shown that miR-291b-3p inhibitor ameliorated hepatic triglyceride (TG) accumulation, reduced fatty acid synthase (FAS) and Sterol-regulatory element binding protein (SREBP-1) activity, which was attributed to the targeting of AMPK by miR-291b-3p^24^. As an energy sensor, AMPK maintains the lipid and glucose homeostasis both at the cellular and the whole body level^26^. We then determined the effect of miR-291b-3p on HFD-induced hyperinsulinemia and hyperglycemia. Notably, hepatic inhibition miR-291b-3p expression improved glucose tolerance, insulin tolerance and pyruvate tolerance. Moreover, ad-miR-291i-treated mice demonstrated higher glucose infusion rates than ad-NC mice. In addition, inhibition of miR-291b-3p reduced glucose production in the medium of cultured NCTC1469 cells. Conversely, overexpression of miR-291b-3p in C57BL/6 J mice caused remarkable impaired glucose tolerance, insulin sensitivity and glucose homeostasis. More importantly, we also found that fasting increased the expression of miR-291b-3p in the liver of C57BL/6 J mice. Consistently, the expression levels of gluconeogenic genes, including G6Pase, PGC-1α and PEPCK, were markedly induced in the fasting state. However, short-term fasting did not change the mRNA level of AMPK in the livers of C57 mice, indicating miR-291b-3p may control glucose production through a different mechanism.

So far, impaired AKT/GSK/FoxO1 activation of cellular pathways transducing insulin signaling has been tightly correlated to hepatic insulin resistance^22^,^23^. Thus, we speculated that aberrant miR-291b-3p expression might lead to abnormal AKT/GSK/FoxO1 phosphorylation in diabetic mouse livers. In line with the metabolic changes, inhibition of hepatic miR-291b-3p substantially activated the AKT/GSK/FoxO1 signaling pathway and ad-miR-291m-treated C57BL/6 J mice exhibited decreased phosphorylation level of AKT/GSK/FoxO1 compared with ad-NC. As a potent regulator of PI3K/AKT signaling, PTEN could negatively regulate the PI3K pathway through dephosphorylating PIP_3_, the second messenger formed by PI3K activation, and then reduced the phosphorylation of AKT^27^,^28^. In ob/ob and db/db mice, knockdown of PTEN in the liver and in the adipose tissue significantly reversed hyperglycemia and reduced blood glucose level^29^. Furthermore, in PTEN−/+ heterozygous mice, insulin sensitivity and glucose tolerance were found to be markedly improved^30^,^31^. We then carried out detailed analysis of PTEN expression changes both *in vivo* and *in vitro*. Of note, inhibition of miR-291b-3p repressed expression of PTEN protein in the liver of HFD-fed mice and NCTC1469 hepatocytes. In comparison, forced expression of miR-291b-3p increased the expression of PTEN in C57BL/6 J mouse livers and NCTC1469 cells. Thus, miR-291b-3p impaired PI3K/AKT signaling mainly by positively regulating PTEN expression in the liver. Although we have demonstrated that AMPK was a direct target gene of miR-291b-3p, no direct interaction between PTEN and AMPK has been reported. Therefore, we further explored another possible target gene of miR-291b-3p that modulated the expression of PTEN, thereby regulating AKT/GSK/FoxO1 signaling pathway.

It was well established the transcription factors of PTEN expression in all cell types^27^. In the present study, we characterized the expression patterns of several proteins including p38MAPK, JNK, Smad2/3 and NF-κB (p50 and p65) that were implicated in modulating PTEN transcription^25^. Notably, we found that miR-291b-3p functions as a negative regulator of NF-κB subunit p65, but not p38MAPK, JNK, Smad2/3 and NF-κB subunit p50. And dual luciferase reporter assay revealed that p65 was a target gene of miR-291b-3p. The negative regulation of p65 to PTEN expression has been identified^19^,^32^,^33^,^34^. In mouse embryo fibroblasts and non-small cell lung cancer cells, suppressing PTEN expression through an NF-κB-dependent pathway prompted cell survival^32^. In adult T-cell leukemia/lymphoma, the reduced N-myc downstream-regulated gene 2 (NDRG2) expression suppressed the expression of PTEN mainly by activating NF-κB signaling, which resulted in the constitutive activation of PI3K/AKT signaling^34^. A similar phenomenon was also observed in our study. We demonstrated that knockdown of p65 augmented the expression of PTEN protein in NCTC1469 cells. More importantly, inhibition of miR-291b-3p partially restored the activation of AKT even in NCTC1469 cells transfected with the specific siRNA targeting p65. Given the important role of AKT in insulin signaling, the activation of AKT by NF-κB subunit p65-mediated repression of PTEN expression might sensitize cells to insulin action in the liver of obese mice. That is, miR-291b-3p indirectly reduced AKT/GSK**/**FoxO1 phosphorylation via the p65/PTEN pathway, thereby contributing to the negative regulating role of miR-291b-3p in insulin signaling.

It would be interesting to know the mechanism that controls the hepatic expression/induction of miR-219b. There are several reports that transcription factors can bind to the promoter regions of specific miRNA genes and activate the transcription of pri-miRNAs, resulting in increased expression of mature miRNAs. For instance, several transcription factors including c-Myc and CTCF have been indicated in the regulation of miR-290 family^35^,^36^. Moreover, epigenetic alterations such as DNA methylation may be involved in controlling miRNA expression. However, in the present study, we did not investigate the mechanism that controls the hepatic expression/induction of miR-291b-3p. It is a limitation in the present study.

**Figure 7:**
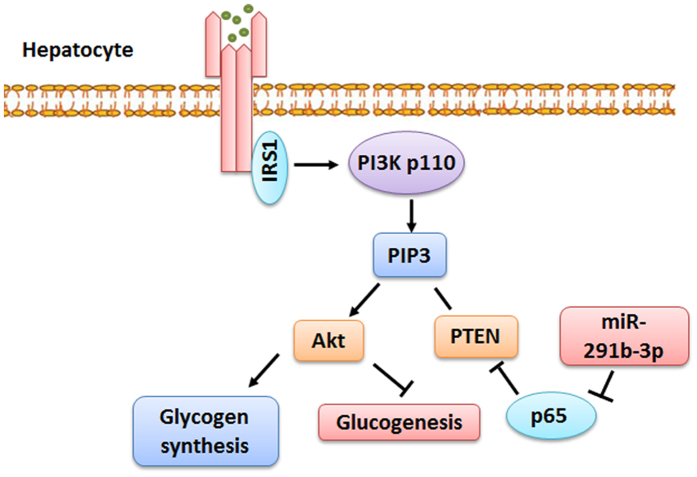
Proposed mechanisms by which hepatic miR-291b-3p regulates insulin signaling and glucose metabolism. MiR-291b-3p targets p65 to regulate PTEN expression, in turn mediated glycogen synthesis and gluconeogenesis in hepatocytes.

In conclusion, as indicated in Fig. 7, we proposed mechanisms by which hepatic miR-291b-3p regulates insulin signaling and glucose metabolism. MiR-291b-3p targeted p65 to regulate PTEN expression, in turn mediated glycogen synthesis and gluconeogenesis in hepatocytes. Our findings indicate the therapeutic potential of miR-291b-3p inhibitor in hyperglycemia and insulin resistance.

## Methods

### Materials

The antibody against β-actin (#4970), p-AKT Ser473 (#4060), AKT (#4691), p-GSK Ser9 (#5558), GSK (#9315), p-AMPK (#8208), PTEN (#9188), p65 (#8242) were purchased from Cell Signaling Technology (Boston, Massachusetts, USA).

### Animal treatment

C57BL/6 J male mice were purchased from the Peking University Health Science Center (Beijing, China). To establish a diet-induced obese model, three-to-four-week-old C57BL/6 J mice were fed a standard chow diet or a high-fat diet (HFD, D12451, 45% kcal from fat, Research Diet, USA, http://www.researchdiets.com/opensource-diets/stock-diets) for 10 weeks in a temperature- (20–24 °C) and humidity-controlled (45–55%) environment. Mice were injected intravenously through the tail vein with adenovirus encoding green fluorescent protein (Ad-NC), miR-291b-3p mimic (Ad-miR-291m) or inhibitor (Ad-miR-291i) at a dose of 1 × 10^9^ plaque-forming units (PFU) in 0.2 ml PBS (0.2 ml/25 g body weight). Mice were sacrificed and tissues were harvested for real-time PCR and Western blot analysis on day 9 after adenovirus injection. For the fasting study, five-to-six-week-old mice were either allowed free access to food or subject to 16 h fast before euthanized for tissue harvest.

All animal experiments were performed in accordance with recommendations in the National Research Council Guide for Care and Use of Laboratory Animals, with the protocols approved by the Animal Ethics Committee at the Beijing Institute of Geriatrics.

### Cell culture

NCTC1469, a murine liver cell line (American Type Culture Collection), was cultured in Dulbecco’s Modified Eagle’s Medium (DMEM) supplemented with 10% (v/v) horse serum (Hyclone, Logan, Utah, USA), 100 units/ml penicillin (Invitrogen, Carlsbad, California, USA), and 0.1 mg/ml streptomycin (Hyclone, Logan, Utah, USA), at 37 °C in a humidified atmosphere with 5% CO_2_.

### Preparation of recombinant adenovirus

Adenovirus vector expressing GFP or miR-291b-3p mimic or inhibitor was generated as previously described^24^,^37^.

### Transient transfection

Firstly, 6 × 10^5^ cells were equally seeded in the 6-well plates. MiR-291b-3p mimic, inhibitor, or miR negative control (NC) (Genepharma, Shanghai, China) were mixed with HiperFect transfection reagent (QIAGEN, Duesseldorf, German) and incubated at room temperature for 10 min according to the manufacturers’ instructions. Then, the complex was transfected into NCTC1469 cells for 48 h.

### RNA extraction and real-time PCR

The total RNA from mouse tissues or NCTC1469 cells was extracted with TRIzol (Invitrogen, Carlsbad, California, USA) according to the manufacturer’s instructions. MiR-291b-3p levels were reversely transcribed using Taq-Man MicroRNA Reverse Transcription Kit (Applied Biosystems, Foster City, California, USA). The expression of miR-291b-3p was normalized to that of the U6 snRNA as previously described^38^. For gene expression analysis, real-time PCR was performed using a BioRad IQ5 instrument and SYBR mix (TransGen Biotech, Beijing, China). GAPDH was used as an internal control. The sequences of the primers are presented in Table 1.

### Western blot analysis

Western blot was performed as described previously^24^,^37^.

### Glycogen content measurement

Glycogen levels were measured in cells or liver tissues using a glycogen assay kit (Nanjing Jiancheng Bioengineering co., Nanjing, China) according to the manufacturers’ instructions.

### Luciferase target assay

The 3′untranslated region (UTR) of p65 containing the predicted binding site for miR-291b-3p was cloned into the pmirGLO (Promega, Madison, Wisconsin, USA) luciferase reporter vector. Details of PCR procedures are performed as previously described^24^,^37^. Transfection was performed with Vigofect transfection Reagent (Vigorous Biotechnology, Beijing, China) according to the manufacturer’s recommendations. Luciferase reporter assays were performed using the Dual Luciferase Reporter Assay System (Promega, Madison, Wisconsin, USA). Renilla activity was used as the normalized parameter.

### Inhibition of p65 expression by RNA interference

Briefly, 1 × 10^5^ cells per well were seeded in a 6-well plate. NF-*κ*B p65 siRNA (sc-29410) or a non-specific siRNA (NC) purchased from Santa Cruz Biotechnology (Santa Cruz, CA, USA) was transfected into NCTC1469 cells for 48 h with HiPerFect transfection reagent (Qiagen) as described above.

### Glucose production assay

The cells were washed five times with PBS and then stimulated with 1 nM insulin (Usbio) for 18 h in L-DMEM containing 10% (v/v) horse serum. Glucose concentrations in the medium were determined using a glucose assay kit (Sigma-Aldrich, St. Louis, Missouri, USA).

### Glucose-, insulin- and pyruvate-tolerance tests (GTT, ITT, PTT)

Mice fasted for 12 h were injected intraperitoneally with D-glucose (2 g/kg), or pyruvate (2 g/kg). For ITT assay, recombinant human insulin (Humulin R, 100 U ml^−1^ stock) was diluted in saline to a concentration of 0.075 U ml^−1^ (1:1330) before injection. Then, insulin was injected intraperitoneally (0.1 ml per 10 g body weight) in mice deprived of food for 12 h. Blood glucose levels were determined from the tail vein at 15, 30, 45, 60 and 90 min using a glucometer (One Touch Ultra; LifeScan Inc.).

### Hyperinsulinemic-euglycemic clamp assay

Briefly, HFD-fed mice were injected with ad-miR-291i or ad-NC for 7 days. Five days before the study, catheters were implanted into a carotid artery and a jugular vein of mice for sampling and infusions, respectively^39^. Insulin clamps were performed on mice fasted for 6 h with a brief modification^40^. The rate of insulin infusion was 8.4 mU/kg/min, and glucose infusion rate was maintained at euglycemic levels between 120–140 mg/dl. The clamp period lasted for 2 h in total, and the data were calculated from the steady state of the clamp starting from the last 20 min of the 2-h clamp. Blood glucose levels were measured at 5-min intervals and 10% glucose was infused at variable rates to maintain euglycemia. All infusions were performed using the microdialysis pumps (CMA/Microdialysis, North Chelmsford, MA).

### Statistical analysis

The data are represented as the mean ± standard error of the mean (SEM). The two-tailed unpaired student’s t-tests were used for comparisons of two groups. The ANOVA multiple comparison test (SPSS 13.0) followed by Turkey post hoc test were used for comparisons of two more groups. *P* < 0.05 was considered to be statistically significant.

## Additional Information

**How to cite this article**: Guo, J. *et al*. Hepatic MiR-291b-3p Mediated Glucose Metabolism by Directly Targeting p65 to Upregulate PTEN Expression. *Sci. Rep.* **7**, 39899; doi: 10.1038/srep39899 (2017).

**Publisher's note:** Springer Nature remains neutral with regard to jurisdictional claims in published maps and institutional affiliations.

## Supplementary Material

Supplementary Information

## Figures and Tables

**Table 1:** The primers used in this study.

Primer name	Sequence
GAPDH Forward	GTCGTGTGAACGGATTTG
GAPDH Reverse	AAGATGGTGATGGGCTTCC
G6Pase Forward	CCTGGTCCAGTCTCACAGGT
G6Pase Reverse	CCTGGTCCAGTCTCACAGGT
PEPCK Forward	GAGAGAACTCCAGGGTGCTG
PEPCK Reverse	CCTTGGAGATGCTGAAAAGC
PGC-1α Forward	CACCAGCCAACACTCAGCTA
PGC-1α Reverse	ACGTCTTTGTGGCTTTTGCT
MiR-291 stem-loop primer	GTCGTATCCAGTGCAGGGTCCGAGGTATTCGCACTGGATACGACACAAAC
U6 stem-loop primer	GTCGTATCCAGTGCAGGGTCCGAGGTATTCGCACTGGATACGACAAATATG
MiR-291 Forward	GCAAAGTGCATCCATTTTGTTTGT
U6 Forward	GCGCGTCGTGAAGCGTTC
Universe revere primer	AAGATGGTGATGGGCTTCC
