# Supplementary material for: Hepatic MiR-291b-3p Mediated Glucose Metabolism by Directly Targeting p65 to Upregulate PTEN Expression
Source: Sci Rep. 2017 Jan 5;7:39899. doi: 10.1038/srep39899 (PMC5214750; doi:10.1038/srep39899)
Supplement: Supplementary Information [file srep39899-s1.pdf]

Hepatic MiR-291b-3p Mediated Glucose Metabolism by Directly Targeting p65 to Upregulate PTEN Expression

Jun Guo[1†], Lin Dou[1†], Xiangyu Meng[1], Zhenzhen Chen[2], Weili Yang[2], Weiwei Fang[1], Chunxiao Yang[1], Xiuqing Huang[1], Weiqing Tang[1], Jichun Yang[2], Jian Li[1*]

[1]The MOH Key Laboratory of Geriatrics, Beijing Hospital, National Center of Gerontology, Beijing, 100730, P. R. China

[2]Department of physiology and pathophysiology, key laboratory of molecular cardiovascular science of the ministry of education, Peking University Health Science Center, Beijing 100191, China

[*] Address correspondence and reprint requests to Jian Li, The Key Laboratory of Geriatrics, Beijing Hospital & Beijing Institute of Geriatrics, Ministry of Health, Beijing 100730, China. E-mail: lijian@bjhmoh.cn.

[†]These authors contributed equally to this work.

Figure legends

Supp. Fig. 1. Full-length blots/gels of the phosphorylation of AKT and GSK in the liver of HFD-fed mice on 7 days after injection of Ad-miR-291i or Ad-NC.

Supp. Fig. 2. Full-length blots/gels are presented and cropping lines are indicated in red color. The phosphorylation of AKT and GSK in the liver of C57BL/6J mice on 7 days after injection of Ad-miR-291m or Ad-NC.

Supp. Fig. 3. Full-length blots/gels are presented and cropping lines are indicated in red color. The phosphorylation of AKT and GSK in the NCTC1469 cells transfected with miR-291b-3p inhibitor (291i).

Supp. Fig. 4. Full-length blots/gels are presented and cropping lines are indicated in red color. The phosphorylation of AKT and GSK in the NCTC1469 cells transfected with miR-291b-3p mimic (291m).

Supp. Fig. 5. Full-length blots/gels for Fig. 5 are presented and cropping lines are indicated in red color.

Supp. Fig. 6. Full-length blots/gels for Fig. 6A and 6B are presented and cropping lines are indicated in red color.

Supp. Fig. 7. Full-length blots/gels for Fig. 6E and 6F are presented and cropping lines are indicated in red color.

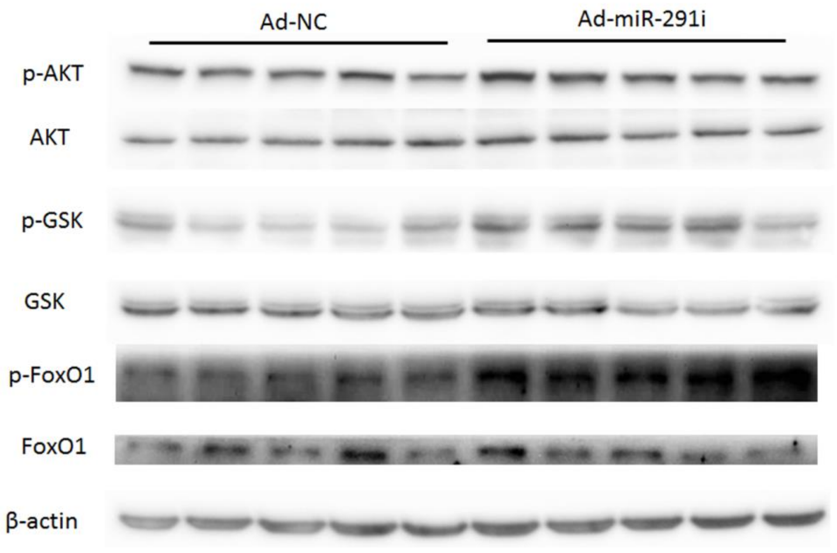

Ad-NC
Ad-miR-291i
p-AKT
AKT
p-GSK
GSK
p-FoxO1
FoxO1
β-actin

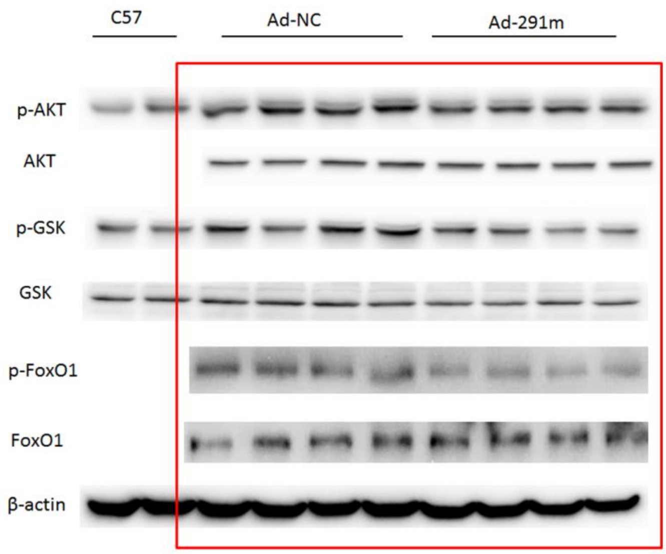
C57
Ad-NC
Ad-291m
p-AKT
AKT
p-GSK
GSK
p-FoxO1
FoxO1
β-actin

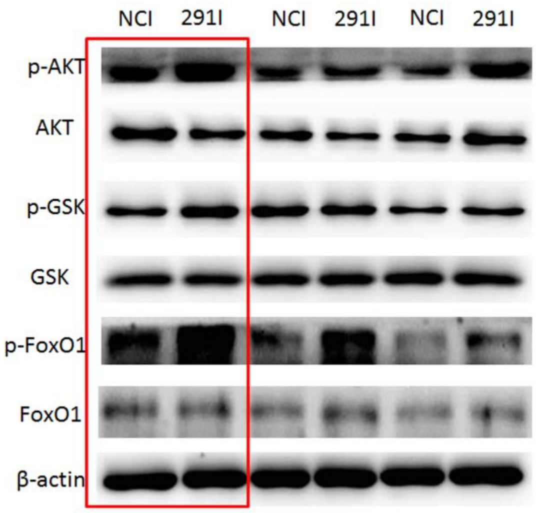
NCI
291I
NCI
291I
NCI
291I
p-AKT
AKT
p-GSK
GSK
p-FoxO1
FoxO1
β-actin

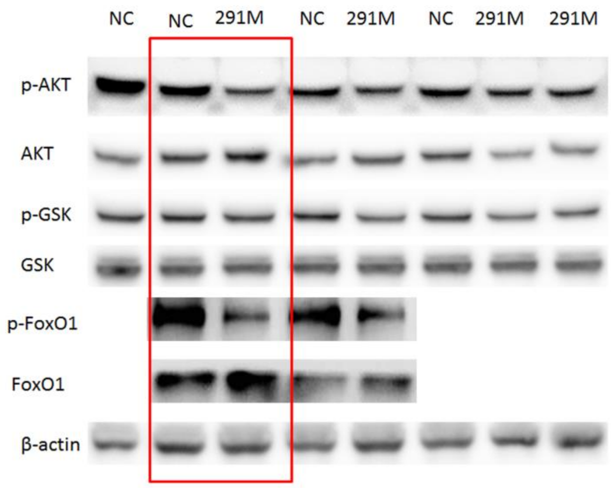

NC
NC
291M
NC
291M
NC
291M
291M
p-AKT
AKT
p-GSK
GSK
p-FoxO1
FoxO1
β-actin

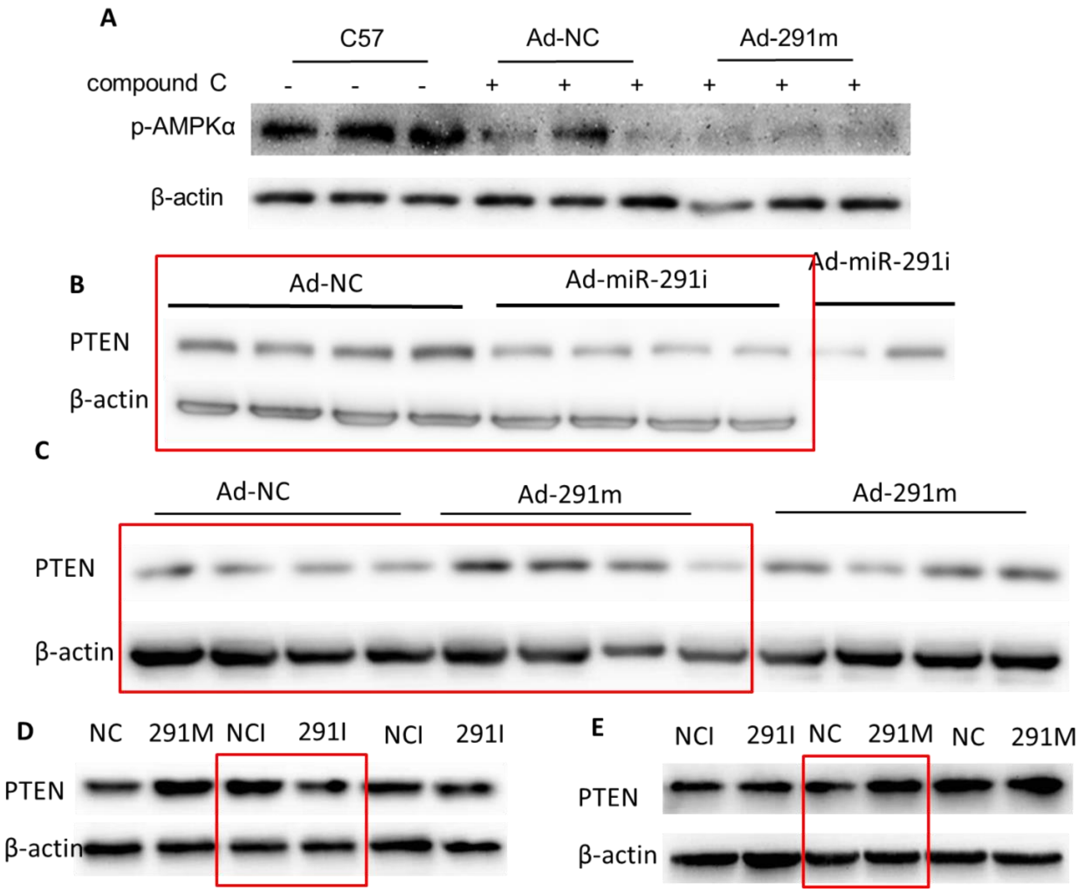

A
C57
Ad-NC
Ad-291m
compound C
- - - + + + + + +
p-AMPKα
β-actin
B
Ad-NC
Ad-miR-291i
Ad-miR-291i
PTEN
β-actin
C
Ad-NC
Ad-291m
Ad-291m
PTEN
β-actin
D
NC 291M NCI 291I NCI 291I
PTEN
β-actin
E
NCI 291I NC 291M NC 291M
PTEN
β-actin

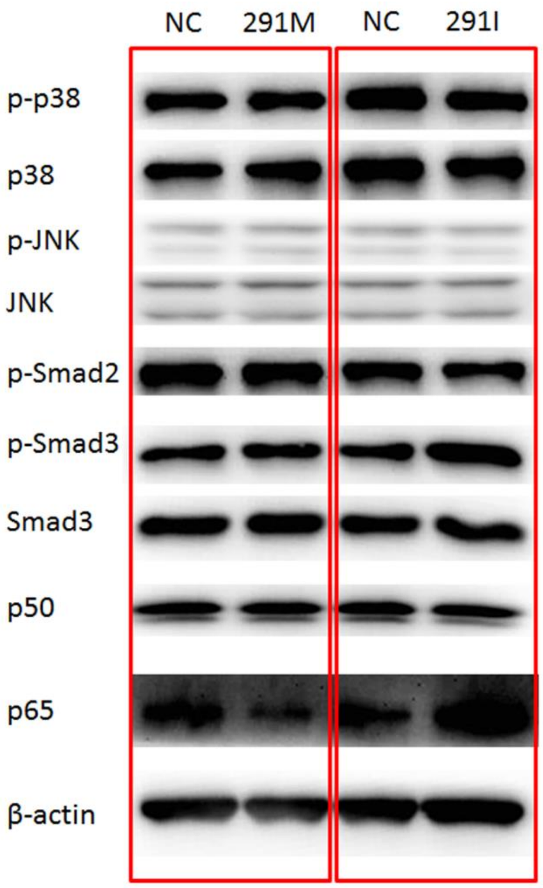
NC
291M
NC
291I
p-p38
p38
p-JNK
JNK
p-Smad2
p-Smad3
Smad3
p50
p65
β-actin

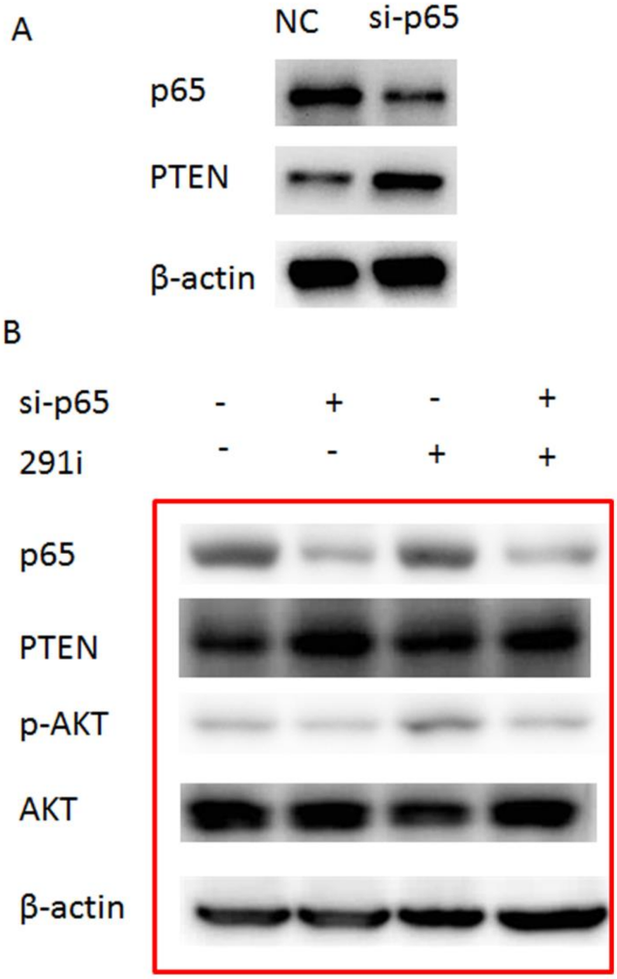
A
NC
si-p65
p65
PTEN
β-actin
B
si-p65 - + - +
291i - - + +
p65
PTEN
p-AKT
AKT
β-actin
